# Patients’ Viewpoints on Using the Sehhaty Mobile Health Application in the Western Region of Saudi Arabia: A Q-Methodology Study

**DOI:** 10.3390/healthcare14193246

**Published:** 2026-10-01

**Authors:** Nabil Alhassani, Abdulrahman Muslihi, Asim Alhejaili, Yasser Allehyani, Sultan Alsahli

**Affiliations:** 1 Department of Health Administration and Hospital, College of Public Health and Health Informatics, Umm Al-Qura University, Makkah 24382, Saudi Arabia; yklehyani@uqu.edu.sa (Y.A.); sssahli@uqu.edu.sa (S.A.); 2 Department of Public Health, College of Nursing and Health Sciences, Jazan University, Jazan 45142, Saudi Arabia; amuslihi@jazanu.edu.sa; 3 Medical-Surgical Nursing Department, Nursing College, Taibah University, Al-Madinah 42353, Saudi Arabia; ahojeli@taibahu.edu.sa

**Keywords:** mHealth, patient portal, Q-methodology, UTAUT, patient viewpoints, digital health, technology adoption

## Abstract

**Highlights:**

**What are the main findings?**

- Patient viewpoints on Saudi Arabia’s national Sehhaty patient portal cluster into two empirically distinct profiles; first, “Cautious Users” and second, “Functionally Engaged Users,” which together account for 51% of the study variance (i.e., variance among the 24 participants’ Q-sorts, not population-level variance).
- The two profiles diverge on perceived functional and clinical value, not on usability, security trust, or digital access, all of which were endorsed by consensus across both groups.

**What are the implications of the main findings?**

- Participants in both profiles did not attribute their Sehhaty use to age, gender, chronic disease status, or urban/rural residence; however, the small purposive P-set does not permit conclusions about demographic predictors of profile membership.
- Deepening engagement is likely to require provider-led demonstration of functional value through concrete features (e.g., secure patient–provider messaging and real-time laboratory result tracking) and care pathway integration, in addition to usability and access-focused interventions.

**Abstract:**

Background/Objectives: The Sehhaty app (v.3.38.0), the Saudi Ministry of Health patient portal, is the principal digital interface between citizens and the national health system. What distinguishes patients who derive functional value from superficial users remains poorly characterized. This study aimed to identify distinct patient viewpoints on Sehhaty use in western Saudi Arabia using Q-methodology grounded in the Unified Theory of Acceptance and Use of Technology (UTAUT). Methods: A 42-statement Q-set was derived from a systematic literature review covering eight extended UTAUT constructs plus usage behaviour. Twenty-four participants, purposively recruited via a matrix stratified by age, gender, chronic disease status, and location, completed online Q-sorts on a forced quasi-normal distribution (−5 to +5), followed by post-sorting interviews. Analysis used KADE v1.2.1 with centroid factor extraction and varimax rotation; principal component analysis only checked the number of factors. Results: Two viewpoints accounted for 51% of the variance among the Q-sorts. Factor 1 (“Cautious Users”) showed high digital readiness and security confidence but limited perceived functional value. Factor 2 (“Functionally Engaged Users”) perceived the application as efficient, care-integrated, and empowering. Fourteen consensus statements confirmed shared endorsement of usability, information clarity, and security trust. Neither viewpoint attributed app use to age, gender, chronic illness, or location, although the small purposive sample precludes demographic inference. Conclusions: Within the sampled viewpoints, the dominant perceived barrier to deeper engagement was limited perceived functional and clinical value rather than usability, access, or security. This exploratory finding informs Vision 2030 digital health policy and warrants larger quantitative confirmation.

## 1. Introduction

The emergence of patient portals and mobile health (mHealth) applications has fundamentally reshaped how patients interact with healthcare systems [1]. A patient portal is a secure online platform (or application) linked to electronic health records (EHRs), which allows patients to view test results, book appointments, manage prescriptions, and communicate with healthcare providers without necessitating a physical visit [2,3]. In the context of rapidly evolving digital health ecosystems, these tools have grown from supplementary conveniences into integral components of modern care delivery [3].

In Saudi Arabia, the national digital health agenda has positioned mHealth at its centre, with the Sehhaty application representing the Ministry of Health’s (MoH) flagship patient-facing digital initiative [4]. Launched to the public during the COVID-19 pandemic, Sehhaty provides Saudi residents with mobile-based access to personal EHRs, appointment scheduling, vaccination records, telehealth consultations, and medication management [5]. Its integration with the national unified digital health services (i.e., the clinical component of the National Platform for Health and Insurance Exchange Services ‘NPHIES’) has made it the primary interface between patients and government healthcare services across the Kingdom [1,5,6]. Currently, the Sehhaty application is increasingly used by nurses in patient education, monitoring, care coordination, and digital follow-up, while doctors use it to support diagnosis, consultation, and treatment planning [7].

Despite its national reach, patient engagement with the Sehhaty app remains uneven. Users frequently cite technical challenges, including navigation difficulties, slow system responses, and intermittent crashes, as factors that undermine reliability and reduce regular use [8]. Patients also report concerns about data privacy and security, although they tend to trust systems provided by the government [8]. More broadly, there is growing recognition that the determinants of patient adoption cannot be reduced to technical features alone; cultural, contextual, and experiential factors play an equally decisive role in shaping whether, and how, patients engage with digital health tools [9,10,11].

Engagement with digital health platforms also needs to be understood within broader patterns of health-service use and digital transformation. Healthcare-seeking behaviour differs across population groups even within a single healthcare delivery system [12], which implies that a universally available national portal will not be experienced or valued uniformly. At the same time, patient-facing platforms are evolving from administrative scheduling tools toward data-driven and artificial intelligence (AI)-supported services [13], raising new questions about the functional value, reliability, and trustworthiness that users expect from such platforms.

The majority of existing research on mHealth adoption in Saudi Arabia has employed survey-based or quantitative designs, which, while valuable for measuring prevalence and associations, are limited in their capacity to reveal the subjective dimensions of patient experience [14,15]. Fewer studies have explored the subjective viewpoints of patients, including the personal meanings, values, and priorities that inform their relationship with digital health platforms. This gap is consequential, as the design of effective patient engagement strategies requires an understanding not only of what patients do, but of why and how they evaluate their digital health experiences [14,15].

To address this gap, the present study used Q methodology, a mixed-methods approach uniquely suited to systematically mapping subjective viewpoints within a research population [16,17,18]. Q-methodology combines the statistical rigour of factor analysis with the interpretive depth of qualitative inquiry to identify and describe the distinct perspectives held by participants about a given topic [19]. It has been applied across health and social sciences, including nursing, healthcare informatics, and digital health, to explore patient experiences, professional attitudes, and policy perceptions [20,21,22]. Unlike surveys, Q-methodology does not aggregate group opinions but instead reveals the diversity of viewpoint structures that coexist within a population [23]. Conventional surveys score each item independently against researcher-defined constructs and summarise responses as averages across respondents, which can conceal qualitatively different configurations of opinion behind similar mean scores. In a Q-sort, by contrast, each participant ranks all statements relative to one another under a forced distribution, so that trade-offs and priorities among usefulness, ease of use, trust, and risk are made explicit; by-person factor analysis then groups participants who share a similar overall configuration. Because most users of a mandatory national platform can be expected to rate usability and security favourably on a rating scale, this holistic, comparative approach is particularly well suited to revealing what actually differentiates Sehhaty users, and to the heterogeneous patient population using the application. Thus, the objective of this study was to explore and categorize the distinct viewpoints of patients regarding the factors that influence their use of the Sehhaty application using Q-methodology in the western region of Saudi Arabia.

## 2. Methods

### 2.1. Overview of Q Methodology

Q-methodology, developed by William Stephenson in 1935, is a structured research method for the scientific investigation of human subjectivity [16]. It guides participants to express their viewpoints through the systematic ranking of a set of statements (the Q-set) about a defined topic, producing a pattern of responses (the Q-sort) that can be analysed statistically to reveal shared viewpoint structures [19,20,21,22,23,24]. The technique is distinct from conventional qualitative methods in that it identifies commonalities in perspective across participants through by-person factor analysis, rather than relying on narrative analysis alone [16]. Its capacity to reveal and compare distinct viewpoints makes it especially appropriate for topics where diverse experiential perspectives are anticipated and theoretically meaningful, as is the case with patient engagement with a nationally standardised mHealth initiative [18,19,20,21,22,23,24,25].

### 2.2. Concourse Development

The concourse refers to the universe of communicable ideas surrounding the research topic [18,23,25]. It was developed through a systematic review of the literature on mHealth use and adoption [23], drawing on the Unified Theory of Acceptance and Use of Technology (UTAUT) as a structuring framework [26,27]. The UTAUT provides a comprehensive framework for understanding the behavioural, social, and contextual drivers of technology adoption [26,27]. The Q-set was structured around the four core UTAUT constructs and four extensions commonly applied in patient-portal research, namely performance expectancy (PE), effort expectancy (EE), social influence (SI), facilitating conditions (FC), behavioural intention (BI), attitude (ATT), anxiety (ANX), and perceived risk (PR) [5,11,16,26]. This grounding in UTAUT allowed the study to link the identified viewpoints to actionable design and policy recommendations for the Sehhaty Application [28].

Two databases (Scopus and Web of Science) were searched for peer-reviewed articles reporting patients’ perceptions of, and facilitators of or barriers to, the use of patient portals, patient-accessible electronic health records, and mHealth applications. Ten peer-reviewed articles published between 2017 and 2024 were retained as statement sources [28,29,30,31,32,33,34,35,36,37]. A total of 242 statements were mined from these sources. After the removal of 76 duplicate and overlapping statements, 166 unique statements were retained and coded into nine categories: the eight extended UTAUT constructs (PE, EE, SI, FC, BI, PR, ATT, and ANX) plus usage behaviour (UB), the outcome construct of the UTAUT model.

To ensure validity, the retained statements were systematically clustered into theoretically grounded themes and sub-categories corresponding to each UTAUT construct, guaranteeing comprehensive coverage of the research question without overlap, repetition, or redundancy [23,38,39]. Each statement was verified against its designated construct to confirm correct categorisation and internal consistency across the Q-set [23]. To strengthen reliability, the draft Q-set was reviewed by five subject-matter experts to assess content coverage of each construct, statement syntax, clarity, and appropriateness, ensuring that all items were unambiguous and consistently interpretable across participants [39,40]. Watts and Stenner [23] emphasise that Q-set statements must achieve full coverage and constancy, conditions that were upheld through this iterative expert review process. The reduction from 166 to 42 statements proceeded in three steps: statements were clustered within each construct; overlapping, repetitive, and redundant statements were merged or removed while ensuring that every construct remained represented; and the expert review refined the wording of the retained statements. A subsequent pilot test, conducted before data collection, further refined the instrument and produced the final Q-set of 42 statements, in line with established Q methodology guidance [23,38,39].

Because participants were Arabic speakers, the Q-set was presented in Arabic; the Arabic wording was prepared by the bilingual research team, and its clarity was checked during the pilot test.

### 2.3. Participants and P-Set

Participant selection in Q methodology, referred to as the P-set, is purposive rather than probabilistic, prioritising diversity of viewpoints over statistical representativeness [17,18,23]. The P-set size is intentionally small and proportionally linked to the number of Q-set statements, as the unit of analysis in Q methodology is the individual Q-sort configuration rather than the number of participants [17,23]. Brown [17] recommends 40 to 60 participants for a Q study, while Watts and Stenner [23] propose a minimum ratio of one participant per two to three statements as a practical guide. Given the 42-statement Q-set in this study, a P-set of 24 participants was determined to be methodologically appropriate.

To structure purposive selection and prevent demographic confounding in the analysis, a P-set matrix was employed. The P-set matrix is a recommended tool in Q methodology for studies involving participants with diverse characteristics, providing a systematic framework that ensures the sample captures the full range of relevant viewpoints within the population of interest [38,39,40,41]. Four demographic characteristics were identified from the literature as significant factors influencing mHealth adoption and were incorporated into the matrix: age (18–30 years; 30 to <40 years; >40 years), gender (male; female), disease condition (chronic; non-chronic), and geographic location (urban; rural) [38]. The combination of these four characteristics yields 24 theoretically distinct participant profiles (3 [age] × 2 [gender] × 2 [disease condition] × 2 [location] = 24 participants), each representing a unique demographic cell within the matrix. The matrix was used as a recruitment guide to maximise the diversity of viewpoints rather than as a fully crossed design, and participants aged over 40 years and those with a chronic disease were under-represented in the final P-set (Table 1 and Table S2). Participants were recruited through gatekeepers in the western region of Saudi Arabia. Eligibility criteria required participants to (1) be registered users of the Sehhaty application, (2) be able to communicate in Arabic, and (3) represent a cell in the P-set matrix. All participants were current registered users; the frequency and duration of their Sehhaty use were not part of the P-set matrix and were not analysed.

### 2.4. Q-Set and Q-Sort Procedure

The 42 statements comprising the Q-set were each placed on individual cards. Participants were asked to sort the cards onto a fixed-distribution grid ranging from −5 (most disagree) to +5 (most agree), with a neutral zone at 0. The study was conducted online, using the Easy HTMLQ platform (v2.0). Participants were informed that there were no correct or incorrect placements. The post-sorting interviews were conducted via Microsoft Teams.

Following each Q-sort, a post-sorting interview was conducted to explore the reasoning behind each participant’s placement of key statements, particularly those ranked at the extremes (±5). These interviews were conducted in Arabic, audio-recorded with participant consent, transcribed verbatim, and translated into English. Thematic analysis of qualitative data was conducted following the phases outlined by Braun and Clarke [42], providing reflective interpretation of each factor.

### 2.5. Factor Analysis and Rotation

Data analysis followed the standard Q methodology sequence: first, construction of a correlation matrix (an inter-correlation matrix) was computed between all 24 Q-sorts, generating a 24 × 24 correlation matrix in which each cell value represents the degree of agreement between two participants’ Q-sort rankings (Figure S1—see Supplementary Material). High positive correlations indicate shared viewpoint orientations; low or negative correlations indicate divergent perspectives [23,43,44]. This matrix constitutes the input data for the by-person factor analysis performed in the next stage.

Second, by-person factor extraction, and factor rotation; factors were extracted using centroid factor analysis (CFA), the extraction approach traditionally preferred in Q-methodology research because it extracts the common variance shared across Q-sorts and leaves the solution open to subsequent rotation [23,45,46]. Principal component analysis (PCA) serves a different purpose: it is a data-reduction technique that partitions the total (common plus specific) variance and yields a single mathematically determined solution. In this study, PCA was therefore not used as an alternative extraction method; it was run on the same 24 × 24 correlation matrix solely as a diagnostic check on the number of factors to retain (eigenvalues and scree plot; Figure S2 and Table S1). All factor loadings, factor arrays, and interpretations reported here derive from the CFA solution [46,47]. This use of PCA as a supplementary check on the number of factors is consistent with established Q-methodology practice [17,23].

The significance threshold for Q-sort loadings at *p* < 0.01 was calculated as 2.58 × (1 ÷ √N), where *N* = 42 is the number of Q-set statements, giving 0.398 (±0.40) [17]. KADE (Ken-Q Analysis Desktop Edition, v1.2.1), the desktop version of the Ken-Q Analysis application, was used for all calculations [48,49]. CFA was the sole extraction method [17,23]. PCA was applied only to check the number of factors to retain [17,23].

Factor retention was guided by five convergent criteria applied simultaneously rather than by reliance on any single criterion [17,23]:

1. Magic Number Seven Criterion. No more than seven factors retained, to avoid over-extraction of small, unstable factors.
2. Kaiser–Guttman Criterion. Eigenvalues > 1.0, indicating that the factor accounts for more variance than a single Q-sort.
3. Two-or-More Significant Loadings Criterion. Each retained factor must have at least two Q-sorts loading significantly on it.
4. Humphrey’s Rule. The cross-product of the two highest Q-sort loadings on a factor must exceed twice the standard error (2 × 0.15 = 0.30).
5. Scree Plot Inspection. Visual identification of the inflection point in the eigenvalue distribution (Figure S2—see Supplementary Material) [50].

Finally, Factor Score Computation—varimax rotation was applied to the retained factors to maximize interpretability [17,23]. The significance threshold for factor loadings was ±0.40, computed using the established formula 2.58 × (1/√N), where *N* = 42 (the number of Q-set statements), corresponding to *p* < 0.01. Q-sorts loading significantly on only one factor (≥±0.40) were designated “pure loaders” and used as defining sorts for their respective factor arrays; confounding sorts (loading significantly on two or more factors) and non-significant sorts were excluded from factor array computation per standard protocol [17,23].

Excluding confounded Q-sorts from the calculation of factor arrays is standard practice because a Q-sort that correlates significantly with more than one factor expresses a blend of viewpoints and would blur the distinctiveness of each factor estimate if it were used to define it [23]. Confounded and non-significant Q-sorts were nevertheless retained in the dataset, are reported in full in Table S2, and were considered in the interpretation because they indicate how far the two viewpoints overlap. A solution with more factors was not adopted to reduce confounding because a third factor did not satisfy the retention criteria (Section 3.2.1).

### 2.6. Ethical Considerations

Ethical approval was obtained from Biomedical Research Ethics Committee, Umm Al-Qura University, prior to data collection (Approval No. HAPO-02-K-012-2025-09-2863, 25 August 2025). All participants provided written informed consent. Participation was voluntary, and participants were informed of their right to withdraw at any time without consequence. No financial remuneration was provided. Data were stored securely and accessed only by the research team.

## 3. Results

### 3.1. Participant (P-Set) Demographic Characteristics

Twenty-four participants who actively used the Sehhaty application were enrolled in the study. The largest age group was 30 to less than 40 years old (*n* = 11; 45.83%), followed by those aged 18 to 30 years (*n* = 10; 41.66%), with the smallest group being those aged above 40 years (*n* = 3; 12.50%). Gender was equally distributed, with 12 male (50%) and 12 female (50%) participants. The majority of participants did not have a chronic disease condition (*n* = 19; 79.16%), while 5 (20.83%) had a chronic condition. Most participants resided in urban areas (*n* = 14; 58.33%), with 10 residing in rural areas (41.66%). Full demographic details are presented in Table 1.

### 3.2. Factor Analysis

#### 3.2.1. Factor Extraction

The five-criterion approach produced a consistent recommendation for a two-factor solution (Table 2). Centroid factor analysis initially extracted seven factors using the Magic Number 7 criterion. Of these, only Factors 1 and 2 satisfied the Kaiser-Guttman criterion (eigenvalue >1: F1 = 10.30, F2 = 1.90). Both factors also met the two-or-more significant Q-sort loadings criterion and Humphrey’s rule (F1: 0.68; F2: 0.36, both exceeding twice the standard error, 2 × SE = 0.30). The Scree test (Figure S2 and Table S1—see Supplementary Material) confirmed a clear elbow at two factors, further supporting the two-factor solution. PCA eigenvalues (F1 = 10.80; F2 = 2.36; F3 = 1.28) corroborated the CFA results; while the PCA eigenvalue for F3 marginally exceeded 1.0, the Q-sort loading and Humphrey’s rule criteria excluded it from the final solution.

##### Factor Rotation and Flagged Q-Sorts

Varimax rotation produced a two-factor solution in which 13 Q-sorts loaded significantly on one of the two factors. Six Q-sorts loaded on Factor 1 and seven on Factor 2. Nine Q-sorts were confounded (loading significantly on both factors), and two were non-significant (loading below ±0.40 on both factors). The significance threshold was ±0.40, calculated as 2.58 × (1 ÷ √42) = 0.398. Factor 1 explained 27% of the study variance, and Factor 2 explained 24%. The combined explained variance of the two-factor solution was 51% (Table S2—see Supplementary Material). In Q-methodology, this figure refers to the proportion of variance in the 24 × 24 Q-sort correlation matrix captured by the retained factors, that is, how much of the similarity among participants’ Q-sorts the two viewpoints account for. It is not an estimate of population-level variance in app use explained by predictor variables, as in regression-based (R-methodology) studies. Solutions accounting for approximately 35–40% or more of the study variance are generally regarded as sound in Q-methodology [23]. Factor characteristics are detailed in Table 3.

##### Factor Correlation and Reliability

The inter-factor correlation between Factor 1 and Factor 2 was 0.5864, indicating a moderate positive relationship between the two viewpoints, which is consistent with the shared baseline of app acceptability reflected in the consensus statements. Both factors demonstrated high composite reliability (F1 = 0.952; F2 = 0.970), indicating strong internal coherence within each viewpoint. The 13 defining Q-sorts represent 54.2% of the P-set. The moderate inter-factor correlation and the nine confounded Q-sorts (37.5%), all of which were provided by participants without a chronic condition and one of which (P17) loaded positively on Factor 1 and negatively on Factor 2 (Table S2), indicate that the two viewpoints share a substantial common core and that many participants combined elements of both (see Section 4.5).

#### 3.2.2. Consensus Statements

Consensus statements represent items ranked at similar positions across both factors, indicating agreement between the two viewpoints regardless of their distinguishing differences. Fourteen consensus statements were identified (Table 4), reflecting four thematic areas: ease of use and information clarity, device compatibility and satisfaction, absence of privacy and security concerns, and rejection of demographic characteristics as self-attributed reasons for use. In the quotations presented throughout the Results, participants P7, P13, P15, P19, P21, and P24 provided defining Q-sorts for Factor 1, and P1, P3, P10, P11, P12, P20, and P22 for Factor 2. The complete factor arrays for all 42 statements, with distinguishing and consensus statements marked, are provided in Table S3 (see Supplementary Material).

##### The App Is Easy to Use

Both factors regarded the Sehhaty app as easy to use, positioning the corresponding statement toward the positive end of the Q sort (statement 7, +2). This shared view was elaborated during the post-sorting interviews, in which participants described how the app removes the burden of contacting call centers and enduring long waits.

*“Unlike before when they used to call… That takes time and effort, and this [app] makes it easier.”*

*(P3)*

Participants further noted that the app enables them to book appointments and access services independently and without delay.

*“I immediately booked through the app… went immediately, and they let me in for my appointment on time.”*

*(P3)*

Features such as appointment notifications were seen as adding a layer of reliability by helping to prevent missed appointments.

*“It gives you a notification if you have an appointment today… not all hospitals call.”*

*(P3)*

Finally, the simple and well-organised interface was described as easy to navigate for users of all ages.

*“The app is easy, its interface is very easy and simple… everything in it is apparent. I know where to go.”*

*(P7)*

##### Reliable Configuration Supported User Satisfaction

Participants across both factors agreed that the Sehhaty app performed well across different devices, placing the statement “The Sehhaty app works well on my different devices like phone or computer” positively (statement 22, +2) and the statement “I enjoy using the Sehhaty app” at a neutral position (statement 27, 0) in both factors. In the post-sorting interviews, participants explained that the app is not an optional convenience but a mandatory tool, which meant that any malfunction became a source of considerable frustration.

*“I feel frustrated when it doesn’t work because booking appointments has to be done through the Sehhaty app.”*

*(P22)*

Users tended to prioritise reliable core functionality over more novel features such as smartwatch compatibility.

*“It doesn’t need to be on smartwatches because not everyone uses these watches.”*

*(P22)*

At the same time, there was a clear aspiration for the app to evolve into a more comprehensive health-monitoring platform.

*“I expect using smartwatches will improve people’s health, especially those with chronic diseases… providing the clinic with data like movement, sleep, and pulse.”*

*(P22)*

Participants also evaluated the app according to how well it aligned with their everyday needs and circumstances.

*“Some [statements] reflect the reality I face in dealing with the app, and others do not apply to daily situations.”*

*(P19)*

##### Clear Instructions and Information Enhance Users’ Understanding

Information provided within the Sehhaty app was consistently viewed as clear and understandable, with both factors placing the relevant statement near the extreme positive end of the Q sort (statement 9, +4). This clarity, in turn, made it easier for participants to learn how to use the app, as reflected in the placement of the related statement (statement 8, +2). During the post-sorting interviews, participants emphasised the value of information that is easy to understand, since it removes barriers to engagement.

*“I find the information on the Sehhaty app clear and understandable.”*

*(P3)*

The Sehhaty App Opens an Opportunity for a Communication Channel

Participants in both factors agreed that the app helped foster a good relationship with their health team or doctors (statement 15, +1). The post-sorting interviews revealed that the app’s layout is sufficiently intuitive that users instinctively know where to go, even without prior exposure or instruction.

*“I opened the app immediately and knew where to go, even though I had no prior experience booking, hadn’t used it before, or seen anyone use it.”*

*(P3)*

The app was seen to succeed precisely because it avoids the confusing layouts that commonly frustrate users of other applications.

*“There are many sites and apps where it takes a while, and you spend time looking for this button and that button.”*

*(P3)*

Finally, a clear visual hierarchy, with key functions prominently placed, was associated with exceptional efficiency.

*“The Sehhaty app, for me, I immediately saw ‘Book Appointments’, opened it, booked, and it took me a minute or two at most.”*

*(P3)*

##### Privacy Was Not a Concern

Both factors indicated that concerns about information privacy did not deter them from using the Sehhaty app. Participants ranked the statements “Security concerns make me hesitant to use the Sehhaty app” (statement 38, −5 and −4) and “I am worried about the privacy of my health information on the Sehhaty app” (statement 35, −5 and −3) toward the negative end of the sort. The post-sorting interviews suggested that participants viewed the app’s government backing as an effective safeguard against data-privacy concerns, to the extent that the possibility of a breach was regarded as almost inconceivable.

*“This is a government app, and a government app would never leak data, never. Who would they share it with?”*

*(P3)*

Participants actively rejected the notion of data insecurity, pointing to what they perceived as a flawless track record and the national context as evidence.

*“Security concerns… make me hesitant to use the Sehhaty app? Uh, because the app is secure, and my information has never been leaked, so I disagree completely with this.”*

*(P3)*

Finally, the information stored within the app was perceived as routine or “superficial,” which further diminished any sense of risk.

*“No, no, there is no fear for the data. Besides, not all data is there, and what is there is superficial data. These apps we have are protected.”*

*(P7)*

##### Availability of Health Information Was Not an Issue

Participants across both factors reported that they were not afraid of losing their personal health information when using the Sehhaty app (statement 34, −4 and −5). The post-sorting interviews indicated that this confidence was grounded in consistently positive experiences free of technical failures.

*“I’ve never entered the app and it froze on me or my data disappeared, even for a short period. So I’ve never felt a problem.”*

*(P1)*

Participants also believed that the app’s permission system reinforced trust by ensuring that only relevant parties could view their information.

*“No one can access it except the patient themselves and the doctor, and this doctor is only the one who gave me an appointment. No other doctor can access it.”*

*(P7)*

In addition, participants equated the app’s security with the robustness of large-scale government systems designed for data permanence.

*“It’s even more logical that programs like this, which save information on a kingdom-wide level, would have no information loss. In fact, in all e-government applications now, data retrieval has become easy.”*

*(P21)*

Overall, there was an absence of perceived risk: participants felt that the combination of protective measures eliminated any fear or hesitation regarding data safety.

*“Of course not, I’m not afraid because it’s protected, and the app is very protected.”*

*(P7)*

##### Controlling Variables

Participants did not attribute their use of the Sehhaty app to chronic illness, gender, or age (statement 39, −3 and −2; statement 40, −4 and −2; and statement 41, −2 and −3). During the post-sorting interviews, several participants went further, questioning the methodological relevance of these factors; some found the very framing of gender as a variable in app usage problematic.

*“Using the Sehhaty app regardless of gender. This question, I feel its structure is weak.”*

*(P21)*

For these participants, health concerns transcend gender, and the app appropriately reflects this universal access.

*“Everyone uses it; it’s not based on gender, because illnesses don’t differentiate between male and female, and neither does app usage.”*

*(P22)*

In their view, the app provides identical functionality and experience regardless of the user’s gender.

The Sehhaty app was consistently regarded as easy to use, removing the burden of call centres and enabling users to book appointments and access services independently and instantly. It was seen to perform well across different devices and has become a mandatory tool, with users prioritising reliable core functionality over more novel features. The information provided within the app was viewed as clear and understandable, helping users navigate with ease and engage without barriers. Privacy was not regarded as a concern, largely because of the app’s government backing, and users expressed strong trust in its security. Finally, participants in both factors did not regard chronic illness, gender, or age as reasons for their use of the app; this reflects participants’ self-attribution rather than a tested association between these characteristics and usage.

#### 3.2.3. Factor 1: Cautious Users

**Figure 1:**
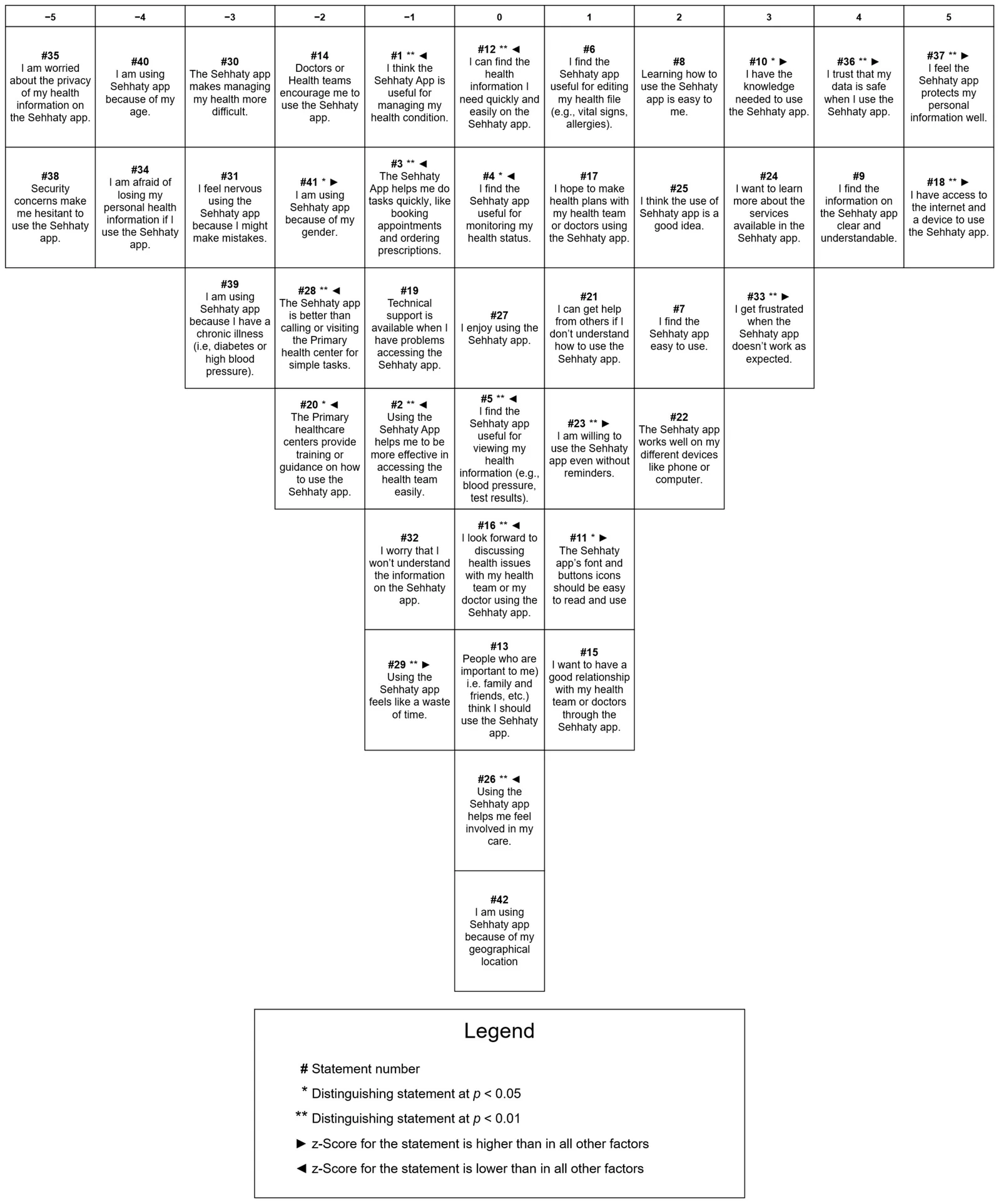
Model Q-sort for Factor 1 (Cautious Users). (*) mark indicates distinguishing statements (* = *p* < 0.05; ** = *p* < 0.01).

Factor 1 (‘Cautious Users’) loaded on 6 participants (P7, P13, P15, P19, P21, and P24), accounting for 27% of the study variance (eigenvalue = 10.30). Participants in this factor were predominantly male (*n* = 4), distributed across age groups, and equally split between chronic and non-chronic conditions. Most resided in urban areas (*n* = 4). Contextual characteristics of participants in Factor 1 are presented in Table 5. The model Q-sort for Factor 1 is shown in Figure 1.

The crib sheet analysis (Table 6) revealed that Factor 1’s most positively endorsed items centred on security and privacy assurance and technical self-efficacy, while its most negatively ranked items were related to functional dependence on the app and performance-based motivation. The two items ranked at +5 were Statement 37 (“I feel the Sehhaty app protects my personal information well”) and Statement 18 (“I have access to the internet and a device to use the Sehhaty app”). Both of these items ranked at 0 for Factor 2, demonstrating the factor’s unique focus on infrastructure readiness and perceived information safety as the primary anchors of app acceptance.

The label “Cautious Users” was chosen to describe these participants’ reserved appraisal of the app’s benefits, not apprehension about technology or data security. Participants defining this factor were confident about security (statements 37 and 36, +5 and +4), access (statement 18, +5), and their own competence (statement 10, +3) and, like Factor 2, did not endorse statements expressing anxiety about using the app (Table S3). Their caution lies instead in withholding endorsement of the app’s functional and clinical value: statements on task efficiency, substitution for primary healthcare visits, access to the health team, and managing a health condition were ranked at −1 to −2 (statements 3, 28, 2, and 1); statements on viewing and finding health information and on discussing health issues through the app were placed at 0 (statements 5, 12, and 16); and frustration when the app does not work as expected was endorsed (statement 33, +3). At the same time, these participants agreed that they would like to learn more about the services available in the app (+3; Table S3), suggesting an open but as yet unconvinced stance.

##### Privacy Was Not an Issue

Drawing on the crib sheet (Table 6), participants felt that their personal information was protected while using the app (statement 37, +5), and they consequently trusted the app to keep their data secure (statement 36, +4).

In the post-sorting interviews, some participants expressed absolute trust in the app’s data protection, conveying unwavering confidence that their information is accessible only to authorised parties.

*“My personal information in the Sehhaty app is very protected. I mean, I am confident about this thing: no one can access my file except the healthcare practitioner and the patient themselves.”*

*(P7)*

The perception that not all personal data is stored within the app further reinforced their comfort with the system, pointing to a sense of selective data visibility.

*“Not everything related to me is in the app. For example, my medical history isn’t there, many test results aren’t there.”*

*(P7)*

Confidence in data safety was consistently expressed alongside ease of use, reflecting a mutually reinforcing sense of trust and usability among users in Factor 1.

*“I trust that my health data is safe… I feel it is protected. Learning to use the Sehhaty app is easy for me.”*

*(P13)*

##### Access and Technical Readiness

Participants in Factor 1 placed considerably greater weight on access and technical readiness than those in Factor 2. They ranked the statement “I have access to the internet and a device to use the Sehhaty app” highly (statement 18, +5), compared with a neutral position in Factor 2 (0), indicating that access is regarded as important for using the app. Interface design was likewise seen as an important motivator in Factor 1: the statement “The Sehhaty app’s font and buttons/icons should be easy to read and use” was ranked at +1 (statement 11) in Factor 1, compared with −1 in Factor 2.

In the post-sorting interviews, many users described the app as entirely straightforward to navigate.

*“I don’t see any difficulty in it.”*

*(P15)*

The app was also seen to provide built-in assistance for users who encounter difficulties.

*“Technical support is available for people in the app.”*

*(P24)*

Healthcare centres were described as actively helping patients in person, creating a bridge between digital and physical support.

*“In the primary care center they help those who need it …. I always see them helping elderly people on how to use the app and benefit from it.”*

*(P24)*

The near-ubiquity of smartphones and internet connectivity was seen to remove barriers to entry.

*“Everyone has a phone in their pocket and has internet, so they don’t need anything else to access the app.”*

*(P24)*

Technical reliability was, however, a salient concern for this group. Factor 1 participants agreed that they become frustrated when the app does not work as expected (statement 33, +3; a distinguishing statement at *p* < 0.01), whereas Factor 2 participants placed this statement at −2. Read together with their descriptions of in-person assistance at primary healthcare centres (P24), this suggests that Factor 1 participants experience malfunction as a meaningful obstacle and retain the primary healthcare centre as a fallback, which is consistent with their reluctance to regard the app as a substitute for calling or visiting the centre (statement 28, −2).

##### Access and Self-Efficacy

Participants in Factor 1 perceived themselves as knowledgeable and capable of using the app without prompting. They ranked the statements “I have the knowledge needed to use the Sehhaty app” (statement 10, +3) and “I am willing to use the Sehhaty app even without reminders” (statement 23, +1) more positively than participants in Factor 2, who placed them at 0 and −1, respectively.

##### Limited Perceived Functional and Care Value

Participants defining Factor 1 consistently assigned lower rankings to statements reflecting functional usefulness and care engagement than those associated with Factor 2, for whom such features were highly salient. For example, while Factor 1 participants ranked “The Sehhaty app is better than calling or visiting the primary health center for simple tasks” negatively (statement 28, −2), the same statement was placed at the highest level by Factor 2 (+5), highlighting a clear divergence in perceived substitution value. A similar contrast emerged for task efficiency: “The Sehhaty app helps me do tasks quickly, like booking appointments and ordering prescriptions” was ranked low in Factor 1 (statement 3, −1) but very highly in Factor 2 (+5).

Post-sorting explanations help to clarify why these participants withheld endorsement of the app’s functional value. First, the clinical content of the app was perceived as incomplete: one participant noted that “not everything related to me is in the app… my medical history isn’t there, many test results aren’t there” and described what is available as “superficial data” (P7). For this participant, the limited content was reassuring from a privacy perspective, but it also meant that the app was not regarded as a meaningful clinical record. Second, the primary healthcare centre remained the reference point for obtaining help, with staff described as assisting patients in person (P24). Third, one participant indicated that some statements “do not apply to daily situations” (P19), suggesting that use of the app is occasional and task-specific rather than embedded in routine health management. Taken together, these accounts portray Sehhaty, from the Factor 1 perspective, as a secure and accessible administrative gateway rather than a tool that changes how care is received.

##### Care Involvement and Communication

Statements relating to care involvement and communication further distinguished the two factors. Factor 1 participants placed neutral to negative emphasis on “Using the Sehhaty app helps me to be more effective in accessing the health team easily” (statement 2, −1) and “Using the Sehhaty app helps me feel involved in my care” (statement 26, 0), whereas both statements were ranked positively in Factor 2 (+2 and +1 respectively). Likewise, statements concerning information access and interaction with healthcare providers—“I can find the health information I need quickly and easily” (statement 12, 0), “I find the Sehhaty app useful for viewing my health information” (statement 5, 0), and “I look forward to discussing health issues with my health team using the Sehhaty app” (statement 16, 0)—were neutral in Factor 1 but strongly positive in Factor 2 (+3 across all three statements). Despite these contrasts, both factors rejected the notion that the app is a waste of time, although Factor 2 did so more emphatically (statement 29, −5 vs. −1 in Factor 1).

Overall, this comparison indicates that Factor 1 users acknowledge access and basic capability but remain unconvinced of the Sehhaty app’s functional and care-enhancing value. This stands in sharp contrast to Factor 2 users, who regard the app as an efficient, engaging, and valuable tool for managing their care.

#### 3.2.4. Factor 2: Functionally Engaged Users

**Figure 2:**
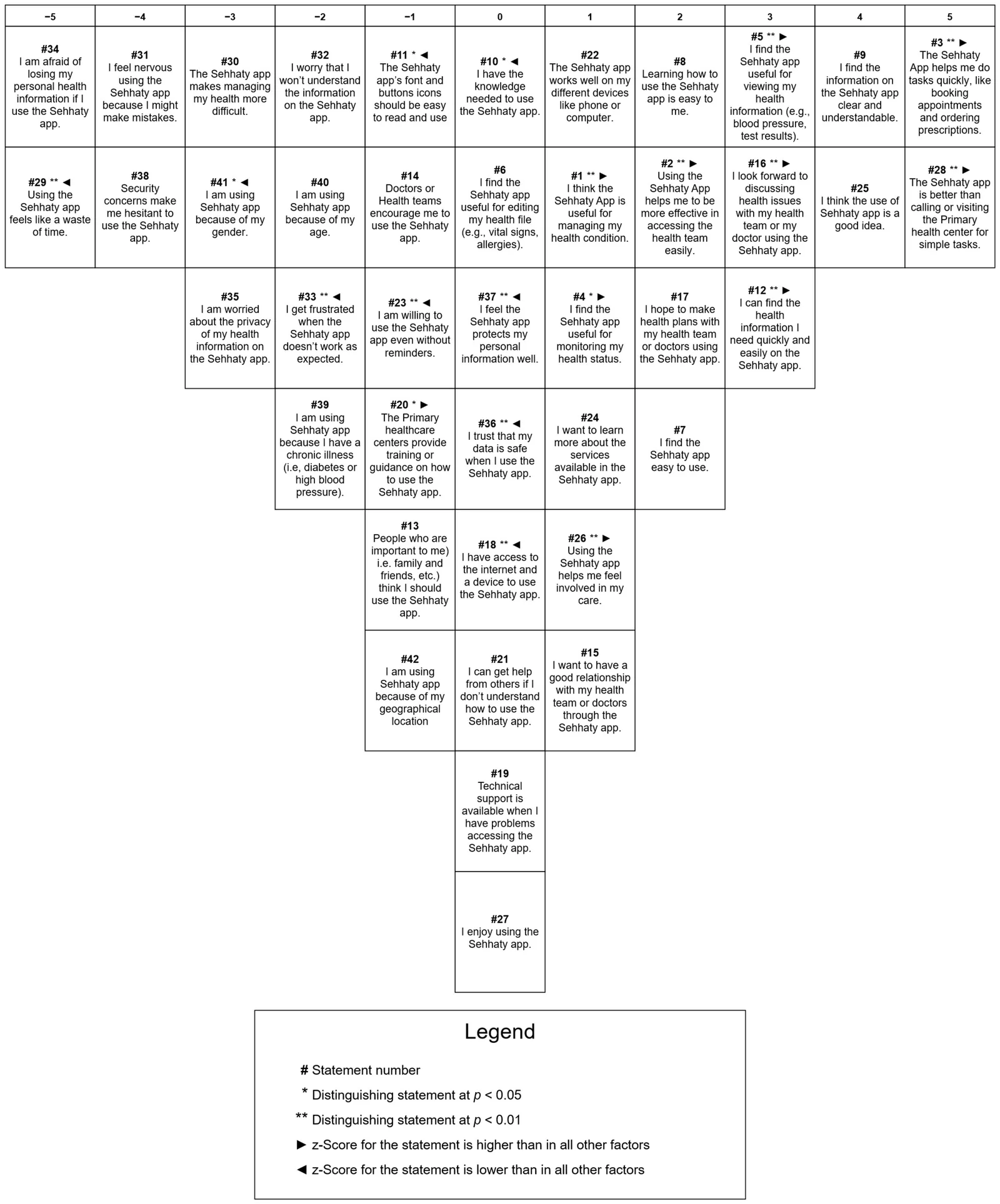
Model Q-sort for Factor 2 (Functionally Engaged Users). (*) mark indicates distinguishing statements (* = *p* < 0.05; ** = *p* < 0.01).

Factor 2 (‘Functionally Engaged Users’) loaded on 7 participants (P1, P3, P10, P11, P12, P20, and P22), accounting for 24% of the study variance (eigenvalue = 1.90). This factor’s defining characteristic is a strong performance-expectancy orientation: participants viewed Sehhaty as a high-value, efficiency-enhancing platform that actively transforms their relationship with healthcare services. Unlike Factor 1, which was anchored in security assurance, Factor 2’s defining items centred on what the app does for the user in functional, clinical, and time-saving terms. The majority of participants in this factor were female (*n* = 5), predominantly aged 18 to 30 years (*n* = 4), mostly without a chronic condition (*n* = 6), and more often from rural areas (*n* = 4). Contextual characteristics are presented in Table 7, and the model Q-sort for Factor 2 is shown in Figure 2.

The crib sheet analysis for Factor 2 (Table 8) confirms that the two items ranked at extreme positive (+5) were Statement 3 (“The Sehhaty app helps me do tasks quickly, like booking appointments and ordering prescriptions”: F2 +5 vs. F1−1) and Statement 28 (“The Sehhaty app is better than calling or visiting the Primary Health Center for simple tasks”: F2 +5 vs. F1−2). These rankings directly contrast with Factor 1, where both statements were placed negatively, confirming that perceived task efficiency and substitution value are the defining hallmarks of this viewpoint. Statement 29 (“Using the Sehhaty app feels like a waste of time”) was ranked at −5 by Factor 2, representing the strongest rejection in this factor and underscoring the group’s conviction in the app’s functional relevance.

##### Strong Endorsement of Efficiency and Practicality

Participants loading on Factor 2 articulated a clear, experience-driven endorsement of the Sehhaty app as a practical and valuable healthcare tool, in sharp contrast to Factor 1. The most defining feature of this factor was its strong emphasis on efficiency and on the substitution of traditional care processes. The two highest-ranked statements were “The Sehhaty app helps me do tasks quickly, like booking appointments and ordering prescriptions” (statement 3, +5 in Factor 2 vs. −1 in Factor 1) and “The Sehhaty app is better than calling or visiting the primary health center for simple tasks” (statement 28, +5 vs. −2), indicating that these users regard Sehhaty as a preferred and superior channel for routine healthcare interactions.

In the post-sorting interviews, participants defining this factor explained their rankings in terms of time saved and the replacement of earlier, more burdensome routes to care. P3 contrasted the app with telephone booking, which “takes time and effort”, and described booking through the app, being seen on time, and completing the task in “a minute or two at most”. Another participant summarised the same experience: “The most useful thing was accomplishing tasks quickly. I can get an appointment, test results are available in less than 24 h” (P10). Appointment notifications were valued because “not all hospitals call” (P3). For these participants, the app was also a necessary channel rather than an optional one: “booking appointments has to be done through the Sehhaty app” (P22). This dependence was accompanied by an expectation that the platform should continue to expand, for example by receiving data from wearable devices so that clinics could view “movement, sleep, and pulse” for people with chronic diseases (P22).

##### Informational Value and Patient–Provider Interaction

Factor 2 participants also placed substantial importance on the app’s role in accessing, understanding, and using health information. Statements such as “I find the Sehhaty app useful for viewing my health information” (statement 5, +3 vs. 0), “I can find the health information I need quickly and easily” (statement 12, +3 vs. 0), and “I find the Sehhaty app useful for monitoring my health status” (statement 4, +1 vs. 0) were ranked positively, reflecting confidence in the app’s informational and monitoring functions. Communication and relational aspects of care were also salient: “I look forward to discussing health issues with my health team or my doctor using the Sehhaty app” (statement 16, +3 vs. 0) and “Using the Sehhaty app helps me to be more effective in accessing the health team easily” (statement 2, +2 vs. −1) emphasised the app’s role in facilitating patient–provider interaction.

##### Engagement and Empowerment in Health Management

Engagement and empowerment further characterised this factor. Statements reflecting involvement in care and condition management—“I think the Sehhaty app is useful for managing my health condition” (statement 1, +1 vs. −1) and “Using the Sehhaty app helps me feel involved in my care” (statement 26, +1 vs. 0)—suggest that Factor 2 users perceive Sehhaty as an active component of their ongoing health management rather than a passive information repository.

##### Neutral View on Enabling Factors and System Assurances

By contrast, enabling factors and system assurances were comparatively less salient for Factor 2. Statements relating to knowledge, access, and privacy—including “I have the knowledge needed to use the Sehhaty app” (statement 10, 0 vs. +3), “I have access to the internet and a device” (statement 18, 0 vs. +5), “I feel the Sehhaty app protects my personal information well” (statement 37, 0 vs. +5), and “I trust that my data is safe” (statement 36, 0 vs. +4)—were placed neutrally, indicating that these aspects are assumed rather than actively valued by this group. Similarly, formal training from primary healthcare centres was not viewed as important (statement 20, −1 vs. −2), suggesting confidence in independent use of the app.

##### Experience of Reliability and Rejection of Time Waste

Notably, Factor 2 participants did not identify with frustration about technical problems, ranking “I get frustrated when the Sehhaty app doesn’t work as expected” negatively (statement 33, −2 vs. +3 in Factor 1). Post-sorting accounts suggest that this reflects a largely trouble-free experience rather than indifference to reliability: “I’ve never entered the app and it froze on me or my data disappeared, even for a short period” (P1). Because these participants depend on the app for routine tasks, reliability remains a precondition of their engagement, and one participant acknowledged that a malfunction is frustrating precisely because booking must be done through the app (P22). Finally, the strongest rejection within this factor was directed at “Using the Sehhaty app feels like a waste of time” (statement 29, −5 vs. −1), underscoring a firm conviction in the app’s value and relevance.

Overall, Factor 2 represents highly engaged, function-oriented users who perceive Sehhaty as an efficient, empowering, and integral digital health platform. This stands in clear contrast to Factor 1 users, whose views are shaped more by access, security, and capability than by perceived functional or care-enhancing benefits.

#### 3.2.5. Integrated Qualitative Interpretation of the Two Viewpoints

Reading the factor arrays alongside the post-sorting interviews suggests that the two viewpoints are separated by three interpretive themes rather than by isolated statement rankings. The first concerns what the app is understood to be. For Cautious Users, Sehhaty is a trusted and accessible gateway whose clinical content is partial, so that it supplements but does not replace contact with the primary healthcare center; for Functionally Engaged Users, it is the default route into care, valued because it removes telephone queues, waiting, and uncertainty about appointments. The second theme concerns the experience of reliability. Cautious Users endorsed frustration when the app does not work as expected and retained in-person services as a fallback, whereas Functionally Engaged Users described uninterrupted performance and consequently did not foreground frustration. The third theme concerns the direction of expectations. Cautious Users expressed interest in learning more about the available services, implying that the app’s clinical functions are not yet visible or relevant to them, whereas Functionally Engaged Users looked ahead to expanded functions such as the integration of wearable data and closer communication with the health team. In participants’ accounts, therefore, feeling empowered by the app was linked to having personally experienced a tangible saving of time and effort, whereas perceiving the app as purely administrative was linked to incomplete clinical content and to the continued availability of face-to-face alternatives.

## 4. Discussion

This Q-methodology study explored patients’ subjective viewpoints toward using the Sehhaty app and identified two distinct perspectives (factors), alongside a set of consensus statements reflecting shared experiences across participants. Overall, the findings indicate that patients commonly perceive the Sehhaty app as easy to learn and use, with clear and understandable information, and they generally do not regard privacy or security as barriers to use. However, patients diverge markedly in how they appraise the app’s functional and clinical value, particularly in relation to its ability to support care management and substitute traditional primary healthcare interactions.

### 4.1. Shared Foundations: Usability, Clarity, and Trust as Baseline Enablers

Across both factors, strong consensus emerged regarding the ease of use, clarity of information, and learnability of the Sehhaty app. Participants consistently ranked statements related to intuitive navigation, understandable information, and low cognitive effort positively, indicating that usability is experienced as a taken-for-granted feature rather than a point of contention. This pattern is consistent with previous mHealth research showing that perceived ease of use and facilitating conditions are strongly associated with intention to use digital health technologies [51,52,53]. Viewed through this lens, the consensus observed in the present study suggests that, for these participants, the Sehhaty app has met baseline acceptance conditions, allowing their evaluations to shift away from usability concerns toward higher-order judgments about functional and clinical value.

Notably, privacy and security concerns were not perceived as barriers by either viewpoint. Statements reflecting fear of data loss, privacy breaches, or security hesitancy were consistently ranked at the extreme negative end across both factors, indicating a shared confidence in the app’s data protection practices. This contrasts with earlier mHealth and patient portal studies conducted in other settings, where privacy and security concerns frequently emerged as dominant inhibitors of adoption [51,52,53]. Within the Saudi context, this finding can be situated within broader national efforts to strengthen digital health governance and establish robust cybersecurity frameworks, which have positioned Saudi Arabia as a global leader in cybersecurity readiness [54,55]. Such a policy and regulatory environment may foster institutional trust in government-led digital health platforms. Consequently, within this regional and cultural context, privacy and security appear to function as hygiene factors, expected and largely taken for granted, rather than as salient drivers differentiating patterns of use. This interpretation should not be generalised to settings with lower institutional trust, to commercial or decentralised health applications, or to populations with more limited digital literacy, where usability and security may remain active barriers.

The near-unanimous rejection of privacy and security fears (statements 34, 35, and 38, ranked between −3 and −5 in both factors) is unusual when set against the international mHealth literature, in which concern about the confidentiality of personal health data is among the most frequently reported reasons for not using, or discontinuing, health apps [56]. Several features of the Saudi context may explain this difference. Sehhaty is a government-operated platform embedded in national infrastructure, including NPHIES and the wider e-government ecosystem developed under Vision 2030, and its use is effectively required for core services such as appointment booking. Users therefore encounter it as an extension of a trusted state institution rather than as a commercial product competing for their data, and participants’ reasoning was explicitly institutional (“a government app would never leak data”; P3). Evidence from other government-led health applications similarly shows that trust in government lowers privacy concerns and raises perceived benefits [57], and that perceived risk may not significantly shape attitudes toward state-provided health apps [58]. By contrast, users of commercial or decentralised health applications must evaluate each provider’s data practices individually, often with limited transparency about secondary data use, and privacy consequently remains a salient barrier in those settings [58]. Two caveats are warranted. First, high institutional trust is not equivalent to an informed appraisal of data governance; one participant regarded the stored data as “superficial” (P7), which suggests that perceived risk may rise as the platform comes to hold richer clinical information. Second, health-system trust, cultural norms regarding public institutions, digital literacy, and perceived privacy are likely to interact, and their relative influence could not be separated in this study.

### 4.2. Factor 1: Cautious Users and the Limits of Perceived Value

Participants defining Factor 1 (“Cautious Users”) demonstrated high confidence in their technical ability, access to digital devices, and trust in data protection, yet simultaneously expressed limited perceived functional and clinical value of the app. Despite acknowledging Sehhaty app’s usability and security strengths, these users remained unconvinced that the app meaningfully improves efficiency, substitutes traditional care pathways, or enhances engagement with healthcare providers. This viewpoint echoes prior mHealth research reporting that digital health applications are often not perceived as substitutes for face-to-face clinical encounters, but rather as complementary tools that coexist alongside traditional care [59,60].

This pattern reflects a capability value gap, whereby digital readiness and technical competence do not necessarily translate into sustained or proactive use. Consistent with earlier studies showing that mHealth is rarely viewed as replacing visits to healthcare professionals, Factor 1 participants assigned lower importance to features related to appointment booking, prescription services, care involvement, and communication [59,60]. For these individuals, the Sehhaty app appears to function primarily as an optional or supplementary resource rather than as an integral component of routine care delivery.

From a theoretical perspective, this viewpoint aligns with extended technology acceptance models, in which performance expectancy and perceived usefulness become more influential than effort expectancy once baseline usability is established [61,62,63]. However, previous mHealth research has also demonstrated that patient engagement is constrained not only by technical design, but by broader socio-technical factors, including digital literacy, user awareness, and contextual readiness [64,65]. Studies have highlighted that usability refinements or interface enhancements alone are unlikely to translate into sustained engagement if users do not perceive clear clinical relevance or experiential benefit. In this light, the present findings suggest that improving technical features in isolation may be insufficient to shift engagement among cautious users unless the app visibly transforms care workflows, strengthens patient–provider interactions, or delivers value that extends beyond existing face-to-face care practices.

Viewed construct by construct, the UTAUT dimensions differed markedly in their capacity to discriminate between the viewpoints. Performance expectancy was the principal axis of differentiation: almost all statements concerning usefulness, task efficiency, and substitution for conventional access were distinguishing statements at *p* < 0.01 (Table S3). Facilitating conditions and perceived risk/trust also discriminated, but in the opposite direction, being foregrounded by Cautious Users and treated as unremarkable by Functionally Engaged Users. By contrast, effort expectancy was a matter of consensus, anxiety statements were not endorsed by either factor, and social influence statements were placed at or slightly below the middle of both arrays, indicating that encouragement from clinicians, family, or friends was not a prominent consideration for either group. A plausible explanation is that, for a mandatory, government-provided platform in a setting with near-universal smartphone access, the constructs that UTAUT treats as drivers of initial adoption (effort expectancy, social influence, and facilitating conditions) approach a ceiling and lose discriminating power, whereas performance expectancy continues to vary because it depends on each user’s care needs and experiences. The findings therefore support the centrality of performance expectancy in the extended UTAUT; extend the model by suggesting that institutional trust operates as a contextual precondition that neutralises perceived risk in state-run platforms; and question whether the UTAUT moderators (age and gender) are perceived by users themselves as relevant to their use, while recognising that this study was not designed to test moderation statistically.

### 4.3. Factor 2: Functionally Engaged Users and Digital Care Integration

In contrast, Factor 2 (“Functionally Engaged Users”) reflects a group that perceives the Sehhaty app as a high-value, efficiency-enhancing, and empowering digital health platform. These users strongly endorsed the app’s ability to replace or outperform conventional healthcare access methods for routine tasks such as booking appointments and managing prescriptions. They also emphasized the importance of rapid access to health information, monitoring personal health data, and facilitating communication with healthcare professionals. This perspective is consistent with prior research demonstrating that digital communication tools, including mobile health applications and telemedicine platforms, can enhance health literacy, support patient education, strengthen self-management behaviors, and improve patient–provider communication, ultimately contributing to better health outcomes [66,67].

It is important to note that the label “Functionally Engaged Users” describes a viewpoint, namely a pattern of perceived usefulness and functional value expressed through the Q-sort, and not objectively measured behaviour. A participant’s Q-sort does not establish how frequently, or for which functions, he or she actually uses Sehhaty, and no app-usage logs were available to this study. The two profiles should therefore be read as differing in perceived functional value rather than in verified functional engagement.

For this group, the Sehhaty app is not merely usable but integrated into everyday health management practices. Engagement is driven by tangible functional benefits and a heightened sense of involvement in care, aligning closely with patient-centered care and digital empowerment frameworks. Previous studies have similarly highlighted the role of mHealth applications in delivering personalized education and ongoing support, which fosters greater confidence and capability in managing health conditions, particularly in chronic disease contexts [68]. Evidence from diabetes- and cardiovascular-focused mHealth interventions further suggests that digital tools can improve self-care behaviors, medication adherence, and clinical indicators when effectively embedded into patients’ daily routines [68,69,70].

For medical-surgical nursing practice specifically, embedding platforms such as Sehhaty into routine ward-based and postoperative care can support nurse-led wound monitoring, structured discharge education, and remote follow-up that extend care beyond the point of discharge [69]. Bibliometric evidence further shows that self-management is now the most prominent research priority in nursing science on mobile applications, underscoring nurses’ central role in translating such digital health tools into everyday bedside practice [70].

This distinction highlights a critical insight that digital health maturity reshapes user priorities. As users become functionally engaged, expectations shift toward efficiency, reliability, and meaningful care integration rather than basic usability or access considerations. System reliability is likely to become increasingly salient as reliance on digital platforms grows. In the present data, however, frustration with malfunction characterised Cautious Users (statement 33, +3) rather than Functionally Engaged Users (−2), who described largely uninterrupted performance; this raises the possibility, which the cross-sectional design cannot test, that negative reliability experiences are one route to a cautious appraisal of the app’s value. This mirrors earlier findings that while simple digital interventions such as SMS-based education can enhance engagement and health outcomes, sustained adoption of more advanced mHealth applications depends on seamless integration with care processes and minimal user burden [69]. Together, these insights suggest that functional value, clinical relevance, and system dependability are central to transforming digital health tools from supportive add-ons into integral components of routine healthcare delivery.

Operationally, demonstrating clinical value implies specific functions rather than general calls for integration. Based on the statements that separated the two viewpoints, priority candidates include: (i) secure two-way messaging between patients and their care team, with defined response times, to make the communication function visible (statements 2 and 16); (ii) real-time release and tracking of laboratory and imaging results with plain-language explanations and trend displays, addressing the perception that clinical content is incomplete (statements 5 and 12); (iii) a more complete longitudinal record, including medical history, medications, and visit summaries across providers through NPHIES; (iv) prescription refill and medication reminder functions linked to pharmacy dispensing; (v) integration of patient-generated data from home devices and wearables with clinician-facing decision support for chronic disease follow-up; and (vi) in-app service-status notices and rapid technical support, given that frustration with malfunction characterised Cautious Users. Provider-side measures are equally important: clinicians and nurses who review portal data and refer to it during consultations signal to patients that the app has clinical consequences. Looking further ahead, patient-facing platforms are increasingly incorporating AI, including symptom triage and conversational assistance [13]. Retrieval-augmented generation, in which the responses of a language model are grounded in retrieved, verifiable sources such as clinical guidelines or the patient’s own record, has been proposed as a way to deliver context-aware patient education and information retrieval, although it raises unresolved concerns about reliability, privacy, explainability, and trustworthy use [71]. The high institutional trust observed in this study is an asset for introducing such functions, but it also places a responsibility on the platform owner to govern them transparently.

### 4.4. Reconsidering Demographic and Clinical Determinism

Across both viewpoints, participants did not attribute their use of the Sehhaty app to age, gender, chronic disease status, or geographic location, as statements linking use to these characteristics were consistently rejected or placed near neutral. This finding concerns how participants themselves explain their use; it does not demonstrate that these characteristics are unrelated to adoption or to viewpoint membership. Q-methodology is designed to identify shared viewpoints, not to estimate demographic predictors, and the present study was neither powered for subgroup comparisons nor based on probability sampling. The finding nonetheless invites caution about purely demographic explanations of digital health uptake and highlights the role of perceived value and integration into care routines. While several studies have reported that younger age, gender, and eHealth literacy are significant predictors of mHealth adoption [72,73,74], the present findings tentatively suggest that within a nationally standardized digital health platform such as the Sehhaty app, experiential factors may outweigh demographic characteristics in shaping sustained engagement. In the Saudi context of widespread smartphone access and high digital infrastructure maturity, user expectations and perceived functional relevance may be at least as important as traditional demographic predictors, a hypothesis that requires testing in adequately powered quantitative studies.

The composition of the P-set further limits what can be said about demographic and clinical characteristics. Although the P-set matrix was designed to sample across 24 demographic cells, the final sample included only three participants aged over 40 years (12.5%) and five with a chronic disease (20.8%). Among the defining Q-sorts, Factor 2 included only one participant with a chronic condition and was predominantly female (five of seven), whereas Factor 1 included three participants with chronic conditions and was predominantly male (four of six). With so few older and chronically ill participants, the study had limited capacity to detect a distinct viewpoint associated with chronic illness, for example one centred on medication management, monitoring, or continuity of care, and the apparent gender and chronic-disease patterning of the two factors may be a product of sample composition. It is also plausible that viewpoints differ between urban and rural residents and between younger and older users in ways that this P-set could not capture. Rural users may value remote booking and teleconsultation more highly because of their distance from facilities, yet may face poorer connectivity; older adults and people with long-term conditions have greater and more complex service needs but may face barriers related to digital literacy and awareness, and uptake of health apps among patients with chronic conditions in Saudi Arabia remains limited [64,75]. Additional or modified viewpoints, such as assisted or proxy use through family members, might therefore emerge in P-sets that deliberately over-sample these groups.

### 4.5. Limitations

The primary limitation of this study is the lack of statistical generalizability due to the small, purposefully selected sample inherent to Q-methodology [44]. While the findings offer deep, idiographic insights into the complexity of patient experiences, they reflect the perspectives of the specific participants and context studied and cannot be directly extended to the broader population. Furthermore, the study was conducted in a region with strong digital infrastructure and institutional trust in government platforms, which may shape viewpoints in ways not transferable to other settings.

Several further limitations should be acknowledged. First, the P-set was small (*n* = 24), purposively recruited through gatekeepers, and drawn from a single (western) region; older adults and people with chronic disease were under-represented. Second, the design was cross-sectional: viewpoints were captured at one point in time, so neither their stability nor any causal relationship between viewpoints, experiences (such as technical problems), and subsequent app use can be established. Third, all data were subjective self-reports obtained through Q-sorting and interviews, which are open to social desirability and recall effects, particularly when a government platform is being evaluated. The frequency and duration of participants’ Sehhaty use were not analysed, and no objective usage data were available; the profiles therefore describe perceived rather than actual engagement, and the contribution of usage intensity to factor differentiation could not be examined. Fourth, only 13 of the 24 Q-sorts (54.2%) defined the two factors, while nine (37.5%) were confounded and two (8.3%) were non-significant. Together with the moderate inter-factor correlation (r = 0.59), this indicates that the two viewpoints are better understood as poles of a continuum built on a shared baseline of acceptance than as mutually exclusive groups. The confounded Q-sorts may represent a hybrid or transitional position, for example, users who rely on the app for booking yet remain unconvinced of its value for clinical communication, or users moving from a cautious to a functionally engaged stance as their experience accumulates; one confounded participant (P17) combined agreement with the Cautious viewpoint with active rejection of the Functionally Engaged viewpoint. Because confounded Q-sorts do not contribute to the factor arrays, this hybrid position is not directly described by our results and deserves dedicated study. Fifth, the Q-set was derived from ten published sources within a UTAUT-based structure; issues outside this framework, such as proxy use on behalf of family members, may not have been represented. Finally, the Q-set and interviews were administered in Arabic and reported in English, and some nuance may have been lost in translation.

## 5. Conclusions

This Q methodology study explored and categorised Saudi patients’ subjective viewpoints on using the Sehhaty mHealth application, identifying two theoretically coherent and practically meaningful viewpoints: Cautious Users and Functionally Engaged Users. Both viewpoints shared a foundation of usability confidence, institutional trust in government data management, and resistance to demographic explanations of app use. The critical distinction between the two viewpoints lay not in usability or security perceptions, which were largely shared, but in perceived functional and clinical value; specifically, whether patients experienced Sehhaty as actively transforming their healthcare engagement or simply facilitating access to it.

Within the sampled viewpoints, limited perceived functional and clinical value, rather than usability, access, or security, emerged as the dominant perceived barrier to deeper engagement. These exploratory findings caution against relying solely on demographic explanations of mHealth adoption and foreground the importance of experiential and value-based factors; they do not establish causal relationships or population-level predictors. For the Saudi MoH, the study offers a structured basis for segmenting patient engagement strategies: moving Cautious Users toward functional engagement is likely to require demonstrating clinical value through concrete functions, such as secure patient–provider messaging, real-time laboratory result tracking, a more complete longitudinal record, and clinical decision-support integration, while maintaining Functionally Engaged Users requires system reliability and expanded clinical functionality. Both goals align with the broader ambitions of Saudi Arabia’s Vision 2030 for a digitally transformed, patient-centred healthcare system.

Future research should examine whether these viewpoints are reproduced in other regions of Saudi Arabia and among specific clinical populations, such as patients with chronic diseases or older adults. In particular, combining Q-methodology with large-scale quantitative surveys, for example by converting the distinguishing statements into a short profile-assignment instrument, and linking responses to objective Sehhaty usage data would make it possible to test whether the two profiles occur at the population level, how prevalent they are across demographic, urban–rural, and clinical groups, and whether profile membership predicts sustained engagement. Longitudinal designs would capture real-world app usage patterns and illuminate how patient viewpoints evolve as experience with the Sehhaty platform accumulates over time.

## Figures and Tables

**Table 1:** Demographic characteristics of the P-set (*N* = 24).

Characteristic	Frequency (*n*)	Percentage (%)
**Age (years)**		
18 to 30	10	41.66
30 to <40	11	45.83
>40	3	12.50
**Gender**		
Male	12	50.00
Female	12	50.00
**Disease Condition**		
Chronic	5	20.83
Non-chronic	19	79.16
**Location**		
Urban	14	58.33
Rural	10	41.66

**Table 2:** Factor extraction criteria summary.

No.	Criterion	F1	F2	F3	F4	F5	F6	F7
1	Eigenvalue (Magic 7)	10.30	1.90	0.78	0.75	0.35	0.76	0.47
2	Kaiser-Guttman (EV >1)	✓	✓	*	*	*	*	*
3	Two or more significant Q-sort loadings	✓	✓	*	*	*	*	*
4	Humphrey’s rule (SE = 0.15; t the double standard Error = 0.30)	0.68 ✓	0.36 ✓	*	*	*	*	*
5	Scree test	✓	✓	*	*	*	*	*

✓ Factor met the criterion; * Factor did not meet the criterion. CFA = centroid factor analysis; EV = eigenvalue.

**Table 3:** Two-factor characteristics after varimax rotation.

Characteristic	Factor 1	Factor 2
Label	Cautious Users	Functionally Engaged Users
Number of participants loaded	6 (25%)	7 (29.2%)
Confounding Q-sorts	9 (37.5%)	
Non-significant Q-sorts	2 (8.3%)	
Explained variance (%)	27%	24%
Average reliability coefficient	0.80	0.80
Composite reliability	0.952	0.970
Standard error of factor Z-scores	0.219	0.173
Inter-factor correlation	0.5864	

**Table 4:** Consensus statements (items ranked similarly across both factors).

Statement No.	Statement	Factor 1	Factor 2
7	I find the Sehhaty app easy to use.	+2	+2
8	Learning how to use the Sehhaty app is easy for me.	+2	+2
9	I find the information on the Sehhaty app clear and understandable.	+4	+4
15	I want to have a good relationship with my health team through the Sehhaty app.	+1	+1
22	The Sehhaty app works well on my different devices (phone or computer).	+2	+1
27	I enjoy using the Sehhaty app.	0	0
30	The Sehhaty app makes managing my health more difficult.	−3	−3
34	I am afraid of losing my personal health information if I use the Sehhaty app.	−4	−5
35	I am worried about the privacy of my health information on the Sehhaty app.	−5	−3
38	Security concerns make me hesitant to use the Sehhaty app.	−5	−4
39	I use the Sehhaty app because I have a chronic illness.	−3	−2
40	I use the Sehhaty app because of my age.	−4	−2
41	I use the Sehhaty app because of my gender.	−2	−3
42	I use the Sehhaty app because of my geographical location.	0	−1

Note: Factor array scores are presented as Q-sort score rankings on the −5 to +5 distribution grid. Statement 41 did not differ between factors at *p* < 0.01 but was flagged as differing at *p* < 0.05 (Figure 1 and Figure 2; Table S3).

**Table 5:** Demographic characteristics of participants in Factor 1 (Cautious Users; *n* = 6).

Variable	Group	*n*	% of Total Sample
Age (years)			
	18 to 30	1	4.17
	30 to <40	3	12.50
	>40	2	8.33
Gender			
	Male	4	16.67
	Female	2	8.33
Chronic Disease Status			
	Chronic	3	12.50
	Non-chronic	3	12.50
Location			
	Urban	4	16.67
	Rural	2	8.33

**Table 6:** Crib sheet of Factor 1 (Cautious Users) compared with Factor 2 (Functionally Engaged Users).

No.	Statement	Factor 1	Factor 2
Items ranked at extreme (+5) in Factor 1			
37	I feel the Sehhaty app protects my personal information well.	+5	0
18	I have access to the internet and a device to use the Sehhaty app.	+5	0
Items ranked higher in Factor 1 than Factor 2			
33	I get frustrated when the Sehhaty app doesn’t work as expected.	+3	−2
36	I trust that my data is safe when I use the Sehhaty app.	+4	0
10	I have the knowledge needed to use the Sehhaty app.	+3	0
23	I am willing to use the Sehhaty app even without reminders.	+1	−1
11	The Sehhaty app’s font and buttons icons should be easy to read and use	+1	−1
Items ranked lower in Factor 1 than Factor 2			
29	Using the Sehhaty app feels like a waste of time.	−1	−5
20	The Primary healthcare centers provide training or guidance on how to use the Sehhaty app.	−2	−1
4	I find the Sehhaty app useful for monitoring my health status.	0	+1
26	Using the Sehhaty app helps me feel involved in my care.	0	+1
1	I think the Sehhaty App is useful for managing my health condition.	−1	+1
2	Using the Sehhaty App helps me to be more effective in accessing the health team easily.	−1	+2
12	I can find the health information I need quickly and easily on the Sehhaty app.	0	+3
5	I find the Sehhaty app useful for viewing my health information (e.g., blood pressure, test results).	0	+3
16	I look forward to discussing health issues with my health team or my doctor using the Sehhaty app.	0	+3
3	The Sehhaty App helps me do tasks quickly, like booking appointments and ordering prescriptions.	−1	+5
28	The Sehhaty app is better than calling or visiting the Primary health center for simple tasks.	−2	+5

Note: Derived from factor arrays. Items ranked at extreme positions or showing the greatest inter-factor difference are presented. Positive values = agreement; negative values = disagreement.

**Table 7:** Demographic characteristics of participants in Factor 2 (Functionally Engaged Users; *n* = 7).

Variable	Group	*n*	% of Total Sample
Age (years)			
	18 to 30	4	16.67
	30 to <40	2	8.33
	>40	1	4.17
Gender			
	Male	2	8.33
	Female	5	20.83
Chronic Disease Status			
	Chronic	1	4.17
	Non-chronic	6	25.00
Location			
	Urban	3	12.50
	Rural	4	16.67

**Table 8:** Crib sheet for Factor 2 (Functionally Engaged Users) compared with Factor 1 (Cautious Users).

No.	Statement	Factor 2	Factor 1
Items ranked at extreme (+5) in Factor 2			
3	The Sehhaty app helps me do tasks quickly, like booking appointments and ordering prescriptions.	+5	−1
28	The Sehhaty app is better than calling or visiting the Primary Health Center for simple tasks.	+5	−2
Items ranked higher in Factor 2 than Factor 1			
5	I find the Sehhaty app useful for viewing my health information (e.g., blood pressure, test results).	+3	0
12	I can find the health information I need quickly and easily on the Sehhaty app.	+3	0
16	I look forward to discussing health issues with my health team using the Sehhaty app.	+3	0
2	Using the Sehhaty app helps me to be more effective in accessing the health team easily.	+2	−1
4	I find the Sehhaty app useful for monitoring my health status.	+1	0
1	I think the Sehhaty app is useful for managing my health condition.	+1	−1
26	Using the Sehhaty app helps me feel involved in my care.	+1	0
Items ranked lower in Factor 2 than Factor 1			
20	The Primary healthcare centers provide training or guidance on how to use the Sehhaty app.	−1	−2
11	The Sehhaty app’s font and buttons icons should be easy to read and use	−1	+1
23	I am willing to use the Sehhaty app even without reminders.	1	+1
10	I have the knowledge needed to use the Sehhaty app.	0	+3
18	I have access to the internet and a device to use the Sehhaty app.	0	+5
37	I feel the Sehhaty app protects my personal information well.	0	+5
36	I trust that my data is safe when I use the Sehhaty app.	0	+4
33	I get frustrated when the Sehhaty app doesn’t work as expected.	−2	+3
Item ranked at extreme (−5) in Factor 2			
29	Using the Sehhaty app feels like a waste of time.	−5	−1

Note: Derived from factor arrays. Items are grouped by ranking position. Positive values = agreement; negative values = disagreement.

## Data Availability

The anonymized Q-sort datasets and factor score matrices generated during this study are available from the corresponding author upon reasonable request. The data are not publicly archived because participant confidentiality commitments in the approved ethics protocol preclude open deposition.

## Supplementary Materials

The following supporting information can be downloaded at https://www.mdpi.com/article/10.3390/healthcare14193246/s1. Figure S1: Correlation matrix of the 24 Q-sorts; Figure S2: Scree plot from the initial principal component analysis; Table S1: Eigenvalues from the initial principal component analysis of the 24 Q-sorts; Table S2: Two-factor solution showing factor loadings and flagging status for all 24 Q-sorts; Table S3: Factor arrays (Q-sort values) for all 42 Q-set statements, with distinguishing and consensus statements.

## Abbreviations

AI	Artificial Intelligence
ANX	Anxiety
ATT	Attitude
BI	Behavioural Intention
CFA	Centroid Factor Analysis
EE	Effort Expectancy
EHR	Electronic Health Record
EV	Eigenvalue
FC	Facilitating Conditions
KADE	Ken-Q Analysis Desktop Edition
mHealth	Mobile Health
MoH	Ministry of Health, Saudi Arabia
PCA	Principal Component Analysis
PE	Performance Expectancy
PR	Perceived Risk
SE	Standard Error
SI	Social Influence
UB	Usage Behaviour
UTAUT	Unified Theory of Acceptance and Use of Technology
