# Supplementary material for: Patients’ Viewpoints on Using the Sehhaty Mobile Health Application in the Western Region of Saudi Arabia: A Q-Methodology Study
Source: Healthcare (Basel). 2026 Oct 1;14(19):3246. doi: 10.3390/healthcare14193246 (PMC13665357; doi:10.3390/healthcare14193246)
Supplement: Supplementary file 1 [file healthcare-14-03246-s001.zip › healthcare-4506688-supplementary.pdf]

Supplementary Materials

Title: Viewpoints on Using the Sehhaty Mobile Health Application in the Western Region of Saudi Arabia: A Q-Methodology Study

The following supplementary materials provide additional analytical detail supporting the factor extraction and rotation decisions reported in the main manuscript. Figure S1 presents the correlation matrix of all 24 Q-sorts, which forms the input for the by-person factor analysis. Figure S2 and Table S1 document the initial principal component analysis (PCA) used to validate the number of factors extracted by centroid factor analysis (CFA). Table S2 presents the complete flagged Q-sort loading matrix, showing each participant's loadings across both factors after varimax rotation, together with their demographic coding. Table S3 presents the factor arrays (Q-sort values) for all 42 Q-set statements, with distinguishing and consensus statements marked.

Figure S1. Correlation Matrix of the 24 Q-Sorts

The correlation matrix presented below shows the Pearson correlation coefficients between all 24 Q-sorts, computed prior to factor extraction. In Q-methodology, this matrix forms the input for by-person factor analysis. High correlations (approaching +1.0) between Q-sorts indicate participants who sorted the 42 statements in a similar pattern — suggesting a shared viewpoint — while low or negative correlations indicate distinct or opposing perspectives. The matrix reveals two broad clusters of co-varying Q-sorts, consistent with the two-factor solution reported in the main text. Pairs of Q-sorts with correlations below the significance threshold ($r < 0.40$) were treated as independent and were eligible for non-significant or confounded classification following rotation.

Correlations between Q sorts

Partici	-P1	-P2	-P3	-P4	-P5	-P6	-P7	-P8	-P9	-P10	-P11	-P12	-P13	-P14	-P15	-P16	-P17	-P18	-P19	-P20	-P21	-P22	-P23	-P24
-P1	100	56	68	47	50	70	35	75	27	66	47	50	45	53	47	66	-27	37	45	54	49	47	49	55
-P2	56	100	58	44	57	61	54	57	32	32	34	46	42	47	48	57	2	44	32	60	34	53	55	47
-P3	68	58	100	56	40	59	34	45	25	54	31	52	35	42	29	50	-24	35	24	48	29	28	57	36
-P4	47	44	56	100	54	50	39	52	43	49	23	32	52	46	63	57	-2	24	33	38	29	42	39	47
-P5	50	57	40	54	100	60	54	60	24	36	40	38	52	56	57	56	24	37	52	52	63	60	41	55
-P6	70	61	59	50	60	100	53	73	26	51	38	48	59	71	57	72	-13	23	61	58	58	49	52	66
-P7	35	54	34	39	54	53	100	32	22	32	19	19	55	44	41	59	41	36	46	34	45	21	37	62
-P8	75	57	45	52	60	73	32	100	22	63	56	41	44	65	62	68	-29	37	50	66	53	56	52	51
-P9	27	32	25	43	24	26	22	22	100	13	15	19	35	31	26	34	10	5	14	20	14	21	13	32
-P10	66	32	54	49	36	51	32	63	13	100	51	39	23	16	34	49	-24	14	33	41	33	33	39	41
-P11	47	34	31	23	40	38	19	56	15	51	100	32	-2	32	20	36	-24	14	19	38	19	26	27	35
-P12	50	46	52	32	38	48	19	41	19	39	32	100	32	31	38	50	-6	24	35	37	26	42	34	31
-P13	45	42	35	52	52	59	55	44	35	23	-2	32	100	58	56	72	37	35	57	42	56	40	45	60
-P14	53	47	42	46	56	71	44	65	31	16	32	31	58	100	52	57	0	24	47	49	41	43	53	54
-P15	47	48	29	63	57	57	41	62	26	34	20	38	56	52	100	68	6	25	48	48	39	64	46	61
-P16	66	57	50	57	56	72	59	68	34	49	36	50	72	57	68	100	13	42	68	45	59	40	48	67
-P17	-27	2	-24	-2	24	-13	41	-29	10	-24	-24	-6	37	0	6	13	100	10	28	-3	28	-2	5	29
-P18	37	44	35	24	37	23	36	37	5	14	14	24	35	24	25	42	10	100	23	26	42	11	43	22
-P19	45	32	24	33	52	61	46	50	14	33	19	35	57	47	48	68	28	23	100	32	51	24	48	52
-P20	54	60	48	38	52	58	34	66	20	41	38	37	42	49	48	45	-3	26	32	100	55	53	54	52
-P21	49	34	29	29	63	58	45	53	14	33	19	26	56	41	39	59	28	42	51	55	100	25	38	57
-P22	47	53	28	42	60	49	21	56	21	33	26	42	40	43	64	40	-2	11	24	53	25	100	42	37
-P23	49	55	57	39	41	52	37	52	13	39	27	34	45	53	46	48	5	43	48	54	38	42	100	34
-P24	55	47	36	47	55	66	62	51	32	41	35	31	60	54	61	67	29	22	52	52	57	37	34	100

Figure S1. Correlation matrix of the 24 Q-sorts, generated using KADE software v1.2.1. Cell values are Pearson correlation coefficients between participant Q-sorts.

Figure S2 and Table S1. Initial Principal Component Analysis (PCA) Eigenvalues

To validate the number of factors retained from centroid factor analysis, an initial principal component analysis was conducted on the same 24 × 24 Q-sort correlation matrix. PCA was used here not as an alternative extraction method but as a diagnostic tool, in accordance with established Q-methodology practice [16,41]. The scree plot derived from this PCA (Figure S2) shows a clear elbow after the second component, providing visual confirmation that a two-factor solution is appropriate. The corresponding eigenvalues are presented in Table S1.

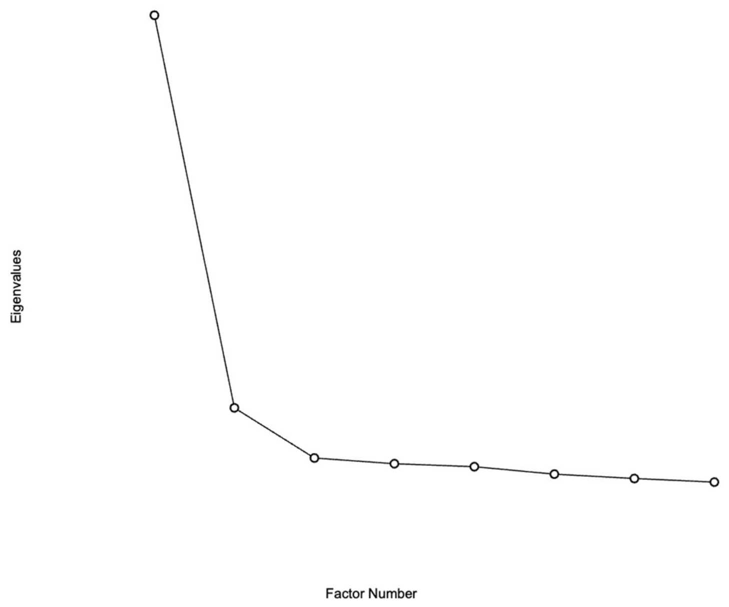

Figure S2. Scree plot from the initial principal component analysis of the 24 Q-sorts. Each point represents one extracted component.

Table S1. Eigenvalues from the initial principal component analysis (PCA) of the 24 Q-sorts.

PCA	Factor 1	Factor 2	Factor 3	Factor 4	Factor 5	Factor 6	Factor 7
**Eigenvalue**	10.7968	2.3576	1.2804	1.1578	1.0942	0.9341	0.8409

PCA was conducted using KADE software v1.2.1 as a validation procedure for the centroid factor analysis solution. Under the Kaiser–Guttman criterion, Factors 1 and 2 yield eigenvalues clearly above 1.0 (10.80 and 2.36). Factors 3–5 return marginal eigenvalues (1.28, 1.16, 1.09); these were not retained because Humphrey's rule and the two-or-more significant loadings criterion, applied to the CFA solution, excluded a third factor. The two-factor solution was confirmed across all five retention criteria reported in the main text.

Table S2. Two-Factor Solution: Q-Sort Loadings and Flagging Status

Table S2 presents the complete loading matrix for all 24 Q-sorts on the two rotated factors following varimax rotation of the centroid factor analysis solution. Each row represents one participant's Q-sort, identified by a coded label encoding their demographic profile (see key below). Factor loadings represent the correlation between each individual Q-sort and the composite factor score. A Q-sort was flagged for a factor when its loading exceeded the significance threshold of ±0.40 (calculated as 2.58 × [1 ÷ √42] = 0.398, where 42 is the number of Q-set statements) and did not load significantly on the other factor. Q-sorts loading significantly on both factors are classified as confounded and excluded from factor score calculations; those not loading significantly on either factor are classified as non-significant.

Of the 24 Q-sorts, 6 were uniquely flagged for Factor 1 (Cautious Users), 7 were uniquely flagged for Factor 2 (Functionally Engaged Users), 9 were confounded, and 2 were non-significant. The confounded Q-sorts are retained in the dataset for transparency but do not contribute to either factor's composite score or interpretation.

Table S2. Two-factor solution showing factor loadings and flagging status for all 24 Q-sorts.

#	Q-sort	Factor 1 loading	Flagging	Factor 2 loading	Flagging
1	P1A1C2G2L1	0.3676		0.7576	Flagged
2	P2A1C2G2L1	0.4538	Confounded	0.5890	Confounded
3	P3A1C2G2L1	0.2518		0.6676	Flagged
4	P4A1C2G2L1	0.5130	Confounded	0.4173	Confounded
5	P5A1C2G2L1	0.6574	Confounded	0.4280	Confounded
6	P6A1C2G1L1	0.5805	Confounded	0.6136	Confounded
7	P7A2C1G2L2	0.6959	Flagged	0.1567	
8	P8A2C2G2L2	0.4296	Confounded	0.7296	Confounded
9	P9A2C2G1L2	0.3253	Not significant	0.1657	Not significant
10	P10A2C1G2L2	0.1745		0.6535	Flagged
11	P11A2C2G2L2	0.0854		0.5507	Flagged
12	P12A1C2G1L2	0.2563		0.5296	Flagged
13	P13A2C2G1L1	0.8344	Flagged	0.1252	
14	P14A2C2G1L1	0.5366	Confounded	0.4484	Confounded
15	P15A1C2G2L1	0.6074	Flagged	0.3857	
16	P16A1C2G2L2	0.7559	Confounded	0.4422	Confounded
17	P17A2C2G1L1	0.5640	Confounded	−0.5731	Confounded
18	P18A2C1G1L1	0.3303	Not significant	0.2777	Not significant
19	P19A2C1G1L1	0.6202	Flagged	0.2502	
20	P20A3C2G1L1	0.3761		0.6194	Flagged
21	P21A3C2G1L1	0.6208	Flagged	0.2748	
22	P22A1C2G2L2	0.3460		0.4983	Flagged
23	P23A2C2G1L2	0.4219	Confounded	0.5140	Confounded

#	Q-sort	Factor 1 loading	Flagging	Factor 2 loading	Flagging
24	P24A3C1G1L2	0.7186	Flagged	0.3195	
	**Explained variance (%)**	**27**		**24**	

Flagging key: Flagged = the Q-sort loads significantly on this factor only and contributes to the factor composite score. Confounded = the Q-sort loads significantly on both factors and is excluded from both factor scores. Not significant = the Q-sort does not load significantly on either factor. Significance threshold: |r| > 0.40 ($p < 0.01$; SE = 0.15). Demographic coding: P = participant number; A = age (1 = 18–30 years; 2 = 31–40 years; 3 = >40 years); C = chronic disease status (1 = chronic; 2 = non-chronic); G = gender (1 = male; 2 = female); L = location (1 = urban; 2 = rural).

Table S3. Factor Arrays for All 42 Q-Set Statements

Table S3 presents the factor arrays, that is, the Q-sort value (−5 to +5) assigned to each of the 42 Q-set statements in the composite (model) Q-sort of each factor, as generated by KADE v1.2.1 and displayed in Figures 1 and 2 of the main text. Statements that distinguish between the two factors are marked with their significance level, and the 14 consensus statements reported in Table 4 of the main text are indicated.

Table S3. Factor arrays (Q-sort values) for all 42 Q-set statements, with distinguishing and consensus statements.

No.	Statement	Factor 1 (Cautious Users)	Factor 2 (Functionally Engaged Users)	Status
1	I think the Sehhaty app is useful for managing my health condition.	−1	+1	Distinguishing (p < 0.01)
2	Using the Sehhaty app helps me to be more effective in accessing the health team easily.	−1	+2	Distinguishing (p < 0.01)
3	The Sehhaty app helps me do tasks quickly, like booking appointments and ordering prescriptions.	−1	+5	Distinguishing (p < 0.01)
4	I find the Sehhaty app useful for monitoring my health status.	0	+1	Distinguishing (p < 0.05)
5	I find the Sehhaty app useful for viewing my health information (e.g., blood pressure, test results).	0	+3	Distinguishing (p < 0.01)
7	I find the Sehhaty app easy to use.	+2	+2	Consensus
8	Learning how to use the Sehhaty app is easy for me.	+2	+2	Consensus
9	I find the information on the Sehhaty app clear and understandable.	+4	+4	Consensus
10	I have the knowledge needed to use the Sehhaty app.	+3	0	Distinguishing (p < 0.05)
11	The Sehhaty app's font and buttons icons should be easy to read and use.	+1	−1	Distinguishing (p < 0.05)

No.	Statement	Factor 1 (Cautious Users)	Factor 2 (Functionally Engaged Users)	Status
12	I can find the health information I need quickly and easily on the Sehhaty app.	0	+3	Distinguishing ($p < 0.01$)
15	I want to have a good relationship with my health team through the Sehhaty app.	+1	+1	Consensus
16	I look forward to discussing health issues with my health team or my doctor using the Sehhaty app.	0	+3	Distinguishing ($p < 0.01$)
18	I have access to the internet and a device to use the Sehhaty app.	+5	0	Distinguishing ($p < 0.01$)
20	The Primary healthcare centers provide training or guidance on how to use the Sehhaty app.	−2	−1	Distinguishing ($p < 0.05$)
22	The Sehhaty app works well on my different devices (phone or computer).	+2	+1	Consensus
23	I am willing to use the Sehhaty app even without reminders.	+1	−1	Distinguishing ($p < 0.01$)
26	Using the Sehhaty app helps me feel involved in my care.	0	+1	Distinguishing ($p < 0.01$)
27	I enjoy using the Sehhaty app.	0	0	Consensus
28	The Sehhaty app is better than calling or visiting the Primary health center for simple tasks.	−2	+5	Distinguishing ($p < 0.01$)
29	Using the Sehhaty app feels like a waste of time.	−1	−5	Distinguishing ($p < 0.01$)
30	The Sehhaty app makes managing my health more difficult.	−3	−3	Consensus
33	I get frustrated when the Sehhaty app doesn't work as expected.	+3	−2	Distinguishing ($p < 0.01$)
34	I am afraid of losing my personal health information if I use the Sehhaty app.	−4	−5	Consensus
35	I am worried about the privacy of my health information on the Sehhaty app.	−5	−3	Consensus
36	I trust that my data is safe when I use the Sehhaty app.	+4	0	Distinguishing ($p < 0.01$)
37	I feel the Sehhaty app protects my personal information well.	+5	0	Distinguishing ($p < 0.01$)
38	Security concerns make me hesitant to use the Sehhaty app.	−5	−4	Consensus
39	I use the Sehhaty app because I have a chronic illness.	−3	−2	Consensus
40	I use the Sehhaty app because of my age.	−4	−2	Consensus
41	I use the Sehhaty app because of my gender.	−2	−3	Distinguishing ($p < 0.05$); Consensus

No.	Statement	Factor 1 (Cautious Users)	Factor 2 (Functionally Engaged Users)	Status
42	I use the Sehhaty app because of my geographical location.	0	−1	Consensus

Notes: Values are the positions of each statement in the composite Q-sort of each factor (−5 = most disagree; +5 = most agree). Distinguishing statements are those whose z-scores differ significantly between the two factors at the level shown; for every distinguishing statement the direction of the difference is given by the two factor values. Consensus = statements reported as consensus statements in Table 4 of the main text (no significant difference between factors at $p < 0.01$); statement 41 is listed as a consensus statement but differs between factors at $p < 0.05$. Statement numbers are shown for the statements cited in the main text (Tables 4, 6 and 8); for the remaining ten statements (–), the wording is given as displayed in the KADE composite Q-sorts (Figures 1 and 2 of the main text), where long statements are abbreviated (…).
